# New Insights into Long Non-Coding RNA *MALAT1* in Cancer and Metastasis

**DOI:** 10.3390/cancers11020216

**Published:** 2019-02-13

**Authors:** Yutong Sun, Li Ma

**Affiliations:** 1 Department of Molecular and Cellular Oncology, the University of Texas MD Anderson Cancer Center, Houston, TX 77030, USA; ysun2@mdanderson.org; 2 Department of Experimental Radiation Oncology, the University of Texas MD Anderson Cancer Center, Houston, TX 77030, USA

**Keywords:** lncRNA, *MALAT1*, metastasis

## Abstract

Metastasis-associated lung adenocarcinoma transcript 1 (*MALAT1*) is one of the most abundant, long non-coding RNAs (lncRNAs) in normal tissues. This lncRNA is highly conserved among mammalian species, and based on in vitro results, has been reported to regulate alternative pre-mRNA splicing and gene expression. However, *Malat1* knockout mice develop and grow normally, and do not show alterations in alternative splicing. While *MALAT1* was originally described as a prognostic marker of lung cancer metastasis, emerging evidence has linked this lncRNA to other cancers, such as breast cancer, prostate cancer, pancreatic cancer, glioma, and leukemia. The role described for *MALAT1* is dependent on the cancer types and the experimental model systems. Notably, different or opposite phenotypes resulting from different strategies for inactivating *MALAT1* have been observed, which led to distinct models for *MALAT1*’s functions and mechanisms of action in cancer and metastasis. In this review, we reflect on different experimental strategies used to study *MALAT1*’s functions, and discuss the current mechanistic models of this highly abundant and conserved lncRNA.

## 1. Introduction

Long non-coding RNAs (lncRNAs) are transcripts that are longer than 200 nucleotides (nt) without protein-coding capacity. Despite the exponential growth in lncRNA publications, our understanding of lncRNA functions and mechanisms is still limited, and outstanding caveats and controversies remain in the current lncRNA knowledge [1,2]. The mechanisms of action of some well-known lncRNAs are currently under discussion [3,4,5,6,7,8,9,10,11]. Questions have also been raised as to whether phenotypes arising from deleting or inactivating a lncRNA gene can be unequivocally attributed to the loss of the lncRNA per se [1]. A recent study revealed opposite effects from the deletion and insertional inactivation of the lncRNA-encoding gene *Haunt*, and remarkably, the gene deletion effect was due to the loss of *Haunt* genomic DNA, which dominated the effect of *Haunt* lncRNA loss [12]. In light of the accumulating evidence for different or opposite phenotypes resulting from different strategies for inactivating the same lncRNA (e.g., *Fendrr*, *Evf2*, and *lincRNA-p21*) in vivo, it has been concluded that genetic rescue experiments, where the lncRNA is re-expressed from an independent transgene, are essential for separating RNA-specific effects from those resulting from the manipulation of the genomic DNA [1]; however, such rescue experiments are generally lacking in the current lncRNA research, especially in cancer studies, making it difficult to interpret many lncRNA results in the cancer field.

Unlike messenger RNAs (mRNAs) and microRNAs (miRNAs), many lncRNAs have poor evolutionary conservation; however, a nuclear lncRNA, metastasis-associated lung adenocarcinoma transcript 1 (*MALAT1*, also known as nuclear enriched abundant transcript 2, *NEAT2*), is exceptionally conserved for lncRNA, and is among the most abundantly expressed lncRNAs in normal tissues [13]. Despite its length (~8 kb in humans and ~7 kb in mice), *MALAT1* is a single-exon gene whose transcript is subject to further processing; for instance, in mice, *Malat1* gives rise to a 7 kb full-length transcript (low expression, nuclear), a 6.7 kb lncRNA (high expression, nuclear), and a 61 nt tRNA-like small RNA (*mascRNA*, exported to the cytoplasm with unknown functions) [14]. At the molecular level, *MALAT1* lncRNA is recruited to nuclear speckles and has been reported to regulate pre-mRNA splicing [13,15]. However, this finding is not supported by *Malat1* knockout mice, which showed normal development and growth and no global difference in alternative splicing [16,17,18]. In addition, *MALAT1* lncRNA is subject to post-transcriptional modifications, such as *N*^6^-methyladenosine (m6A) [19] and 5-methylcytosine (m5C) [20], but the functional consequences of these modifications remain unknown.

Originally, *MALAT1* was identified as a prognostic marker for poor clinical outcomes (overall survival and metastasis-free survival outcomes) in patients with early-stage non-small cell lung cancer [21]. To date, there are more than 800 publications related to *MALAT1* (the PubMed search word “MALAT1” generated 809 results as of 13 February, 2019) and many of them reported a role of *MALAT1* in cancer, making *MALAT1* one of the most studied lncRNAs. Intriguingly, different studies yielded conflicting results about *MALAT1*’s functions and mechanisms of action. In this review, we discuss the progress and controversies in *MALAT1* research, and reflect on the approaches and experimental design used for lncRNA studies.

## 2. Does *MALAT1* Regulate Alternative pre-mRNA Splicing and Global Gene Expression?

By performing RNA fluorescent in situ hybridization and protein immunofluorescent staining, Hutchinson et al. found that *MALAT1* lncRNA co-localizes with SC35 nuclear speckles, structures involved in pre-mRNA processing [13]. Subsequently, based on small interfering RNA (siRNA) knockdown results from cultured cell lines, *MALAT1* was identified as a nuclear-retained regulatory RNA that interacts with the serine/arginine-rich family of splicing factors, affects the distribution of splicing factors in nuclear speckle domains, and regulates alternative splicing of pre-mRNAs [15]. Moreover, by using the CHART-seq technology, West at al. identified hundreds of *MALAT1*-binding sites in human cells and most of these sites are on actively transcribed genes, indicating that *MALAT1* might be involved in regulating gene transcription [22]. However, these effects were absent in genetically engineered mouse models lacking *Malat1* expression [16,17,18].

**Figure 1:**
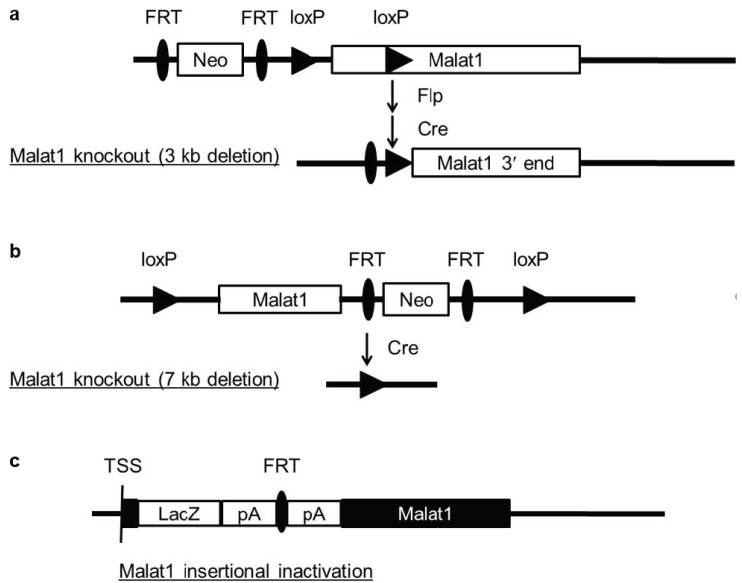
Different strategies used to generate *Malat1* knockout mice. (**a**) A 3 kb genomic region encompassing the 5′ end of *Malat1* and its promoter was deleted. (**b**) The full-length *Malat1*, including 250 bp upstream of the transcriptional start site and 321 bp downstream of the 3′ end of *Malat1*, was deleted. (**c**) The β-galactosidase gene (*lacZ*) with polyadenylation sequences (pA) was inserted 69 bp downstream of the transcriptional start site (TSS) of *Malat1*.

In 2012, three *Malat1* knockout mouse models, generated by different strategies, were reported by independent groups. Zhang et al. removed a 3 kb genomic region encompassing the 5′ end of *Malat1* and its promoter (Figure 1a) [16]. Eissmann et al. deleted the entire 7 kb mouse *Malat1* gene (Figure 1b) [17]. Nakagawa et al. disrupted the *Malat1* gene by inserting a transcriptional terminator (*lacZ* and the polyadenylation sequences) 69 bp downstream of the transcriptional start site of *Malat1* (Figure 1c); similar to the two gene deletion strategies, this insertional inactivation approach also abrogated *Malat1* RNA expression in mice, as gauged by Northern blot analysis and in situ hybridization [18]. Surprisingly, none of these three models showed phenotypes, and loss of *Malat1* in mice did not affect global gene expression, nuclear speckles, or alternative pre-mRNA splicing [16,17,18], which argues against the in vitro siRNA knockdown results [13,15,22]. This discrepancy suggests that in vitro findings could be cell line-specific, or rely on specific experimental settings and approaches. Alternatively, *Malat1* may have stress-dependent functions in vivo. It is also possible that additional factors compensate for the effects of *Malat1* loss in mice. These possibilities warrant further investigation.

## 3. Is *MALAT1* a Metastasis Promoter or a Metastasis Suppressor?

*MALAT1* expression has been shown to be either upregulated or downregulated in human cancers. On one hand, upregulation of *MALAT1* was reported in lung cancer, hepatocellular carcinoma, breast cancer, and colorectal carcinoma, which has been extensively reviewed previously [23,24,25,26]. On the other hand, several studies showed that the expression of *MALAT1* is downregulated in glioma [27], colorectal cancer [28], and breast cancer [29,30]. Previous in vitro and xenograft studies demonstrated that *MALAT1* promotes cell proliferation, migration, tumor growth, metastasis, and chemoresistance [31,32,33,34,35,36,37]. In contrast, other studies reported that *MALAT1* inhibits cell proliferation, tumor growth, invasion, and epithelial-mesenchymal transition (EMT) [27,28,30,38,39,40].

As mentioned above, one of the three *Malat1* knockout mouse models harbors a 3 kb deletion of *Malat1*’s promoter and its 5′ end [16]. After breeding these mice to a metastasis-prone transgenic model of breast cancer, MMTV (mouse mammary tumor virus)-PyMT (polyomavirus middle T antigen) mice [41], Arun et al. observed a reduction of lung metastases [42]. Notably, despite no difference in mammary tumor size, *Malat1* gene-deleted PyMT tumors were liquid-filled and much more differentiated with a drastically increased cystic phenotype [42], which might underlie the metastasis reduction observed in this model. In addition, after treating MMTV-PyMT mice with *Malat1* antisense oligonucleotides (ASOs), Arun et al. also observed increased cystic areas in primary tumors and decreased lung metastases [42]. However, unlike genetic deletion, ASO treatment significantly decreased the tumor volume (by ~50%), suggesting that these ASOs have extra effects other than knocking down *Malat1*. Unfortunately, the molecular mechanisms underlying the *Malat1* gene deletion and ASO effects remain unknown.

In stark contrast, after mice with targeted insertional inactivation of *Malat1* [18] were bred to MMTV-PyMT mice of a C57BL/6 strain, our group found that the transcriptional inactivation of *Malat1* induced a striking increase in the number of visible metastatic nodules and in the number and area of metastatic foci in the lungs [29]. Importantly, the metastatic-promoting effect of *Malat1* insertional inactivation, which contradicted the *Malat1* genomic deletion effect, was completely reversed by the genetic add-back of *Malat1* achieved via breeding to mice with targeted transgenic expression of *Malat1* from the *ROSA26* locus [29]. Consistently, after MMTV-PyMT mice on an FVB background were crossed to the *Malat1* transgenic mice on an FVB background, overexpression of *Malat1* markedly suppressed lung metastasis [29]. In contrast to the gene deletion model [42], our group found that *Malat1* wild-type, *Malat1*-inactivated, and *Malat1*-overexpressing PyMT tumors showed similar degrees of cystic areas and high-grade carcinoma areas [29]. Interestingly, insertional disruption of *Malat1* significantly elevated the percentages of circulating tumor cells (CTCs) in the peripheral blood of MMTV-PyMT mice, which was also reversed by restoration of *Malat1* expression. Furthermore, CRISPR (clustered regularly interspaced short palindromic repeats)-Cas9-mediated knockout of *MALAT1* (~650 bp deletion of the 5′ end) in the MDA-MB-231 breast cancer cell line promoted cell migration and invasion in vitro and lung metastasis in vivo, which could be reversed by ectopic expression of mouse *Malat1*. Conversely, overexpression of *Malat1* in LM2 human breast cancer cells and in 4T1 mouse mammary tumor cells led to a pronounced reduction of their lung metastatic ability in experimental metastasis assays [29]. Taken together, targeted inactivation, restoration (genetic rescue), and overexpression of *MALAT1* in multiple in vivo models suggest that the lncRNA *MALAT1* suppresses breast cancer metastasis.

## 4. Experimental Dissection of *MALAT1* and Other lncRNAs

What led to inconsistent conclusions about *MALAT1*’s function? While it remains to be determined whether this is dependent on different cell/tissue types, cancer types, and genetic backgrounds, we can carefully examine the experimental settings and approaches that have been used to study *MALAT1* and other lncRNAs; this offers important lessons. In fact, substantially different or opposite phenotypes arising from different strategies (e.g., gene deletion, insertional inactivation, CRISPR-Cas9, and RNAi) for inactivating the same lncRNA are not uncommon.

In mice, genetic deletion of the lncRNA *Fendrr* resulted in lung and gastrointestinal tract defects [43], whereas transcriptional terminator insertion led to heart and body wall defects [44]; the defects caused by insertional inactivation were rescued by a *Fendrr* transgene [44]. Moreover, RNAi experiments showed that the lncRNA *Evf2* is important for activating *Dlx5/6* expression [45], but transcriptional terminator insertion in mice caused the opposite effect on *Dlx5/6* expression [46]; the effect caused by insertional inactivation could be rescued by *Evf2* expression from a separate transgene [47]. Strikingly, a recent study reported opposing effects from the lncRNA *Haunt* gene deletion and insertional inactivation [12]. The *Haunt* genomic locus contains enhancers for *HOXA* genes, while *Haunt* lncRNA inhibits *HOXA* expression by binding to chromatin [12]. Yin et al. showed that CRISPR-Cas9-mediated large deletion of the *Haunt* genomic locus attenuated *HOXA* gene activation during retinoic acid-induced embryonic stem cell differentiation, whereas minimal disruption of genomic sequences, such as insertional inactivation by CRISPR knockin, abrogated *Haunt* transcription and upregulated *HOXA* expression [12]. Notably, *Haunt* cDNA was unable to “rescue” the deletion phenotype [12], suggesting that the *Haunt* genomic deletion effect dominated the effect of *Haunt* lncRNA loss. These and other studies strongly demonstrate the importance of rescue experiments.

Two excellent reviews discussed considerations when investigating lncRNAs in general [1,2]. The vast majority of *MALAT1* reports are based on siRNA or short-hairpin RNA (shRNA) experiments, and a few studies [42,48,49] used ASOs. However, nuclear RNAi is not fully established and the RNAi approach can be problematic for nuclear RNAs. Silencing a nuclear RNA by siRNA or shRNA requires nuclear Ago2 and other RNAi factors [50]. It has been shown that subcellular localization of Ago2 depends on cell/tissue types and genetic backgrounds [51]. 

If a cell line or tissue does not have nuclear Ago2, the specificity of the *MALAT1* siRNA or shRNA should be questioned. It should be noted that antisense RNAs can have substantial non-specific effects, and that an alarming and growing number of claimed anticancer targets have been invalidated due to recent proof for off-target effects of previously used antisense RNAs and chemical inhibitors [52]. For example, MELK was previously identified as a kinase required for tumor cell survival and proliferation in several cancer types. RNAi and small-molecule inhibitors of MELK demonstrated anticancer efficacies in many studies, and one of the MELK inhibitors, OTS167, entered several clinical trials. Recently, however, multiple independent studies [53,54,55,56] demonstrated that these results were caused by off-target effects, thereby invalidating MELK as an anticancer target reported by many groups. For these reasons, it is crucial to rule out off-target effects of antisense RNAs by genetic add-back of RNAi-resistant mutants and by multiple loss-of-function approaches. Unfortunately, so far no publication has demonstrated the specificity of the *MALAT1* siRNA, shRNA, or ASO by rescue experiments or by *MALAT1* knockout cells.

Previous cell culture and xenograft studies showed contradictory effects of *MALAT1* on cancer cell growth, proliferation, and invasion [27,30,31,32,38,39,40]. With regard to genetically engineered mouse models, opposite phenotypes were also observed. Whereas genetic deletion of *Malat1* in MMTV-PyMT mice inhibited lung metastasis [42], our group found that targeted insertional inactivation of *Malat1* promoted lung metastasis in the PyMT mouse model [29]. It should be noted that we were able to reverse the insertional inactivation phenotype by genetic add-back of *Malat1* using a targeted *Malat1* transgenic model [29], which suggests that the metastasis-promoting effect of *Malat1* inactivation was due to the loss of *Malat1* lncRNA.

Why did the two different *Malat1* knockout mouse models show different phenotypes? As mentioned above, the *Haunt* lncRNA gene deletion effect has been attributed to the loss of the *Haunt* genomic DNA, which dominated the effect of *Haunt* RNA loss [12]. It is possible that the similar scenario applies to the *Malat1* genomic locus versus *Malat1* lncRNA, although the experimental evidence for this hypothesis is lacking at present. Notably, the *Malat1* genomic deletion model showed significant upregulation of *Malat1*’s 12 adjacent genes [42]; in contrast, the *Malat1* insertional inactivation model showed no changes in expression levels of these neighboring genes both in normal tissues and in mammary tumors [29]. It remains to be determined whether this is the reason for the different phenotypes of the two *Malat1* knockout mouse models. Among the concerns about deletion of lncRNA genomic loci is that large deletions may eliminate regulatory elements for other genes or destroy long-range genomic interactions.

As mentioned above, the *Malat1* gene generates several transcripts with different expression levels and localizations. It should be noted that all three different strategies used to generate *Malat1* knockout mice eliminated all *Malat1* transcripts including the uncharacterized transcripts, and that our group used full-length *Malat1* to restore its expression in *Malat1*-defecient mice and in *MALAT1*-knockout human cells. Among the transcripts derived from the *Malat1* gene locus, the nuclear lncRNA *Malat1* is the predominant form and is expected to be the functional form. Nevertheless, functional dissection of different transcripts warrants future studies.

## 5. Mechanistic Models of *MALAT1* in Cancer and Metastasis

LncRNAs function through binding to other RNA, genomic DNA, or protein. Specifically, a lncRNA can serve as a scaffold that keeps proteins together, as a guide that helps recruit proteins to specific genomic DNA sequences, or as a molecular decoy (also called “sponge”) for proteins and other RNAs. In this section, we discuss several molecular mechanisms by which *MALAT1* regulates tumor progression and metastasis.

### 5.1. MALAT1 Serves a Competitive Endogenous RNA (ceRNA)

**Figure 2:**
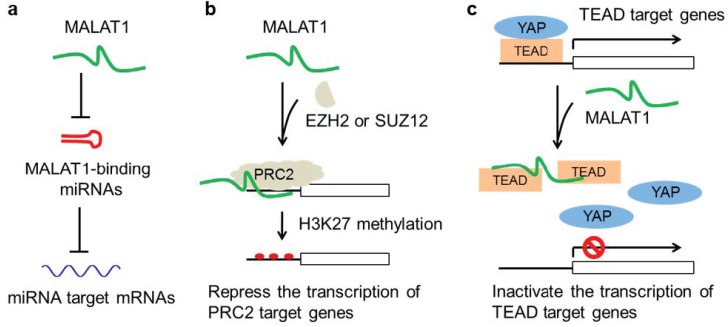
Mechanistic models of *MALAT1* in cancer and metastasis. (**a**) *MALAT1* sponges miRNAs, leading to repression of miRNA target mRNAs. (**b**) *MALAT1* binds PRC2 components and recruits PRC2 to target gene loci. (**c**) *MALAT1* binds, sequesters, and inactivates TEAD.

*MALAT1* is a long and highly abundant lncRNA that contains many putative binding sites of miRNAs. A number of studies reported that *MALAT1* functions through sponging miRNAs, including miR-145 [57], miR-1 [58], miR-202 [59], miR-200c [60], miR-206 [61], miR-204 [62], and so on. In these studies, the authors typically showed that siRNA-mediated knockdown of *MALAT1* in cancer cell lines resulted in a certain phenotype, such as proliferation, migration, invasion, chemosensitivity, or radiosensitivity, followed by luciferase assays to demonstrate the existence of the miRNA-binding site on *MALAT1*. Then, functional experiments demonstrated that the miRNA and its target gene mediate the effect of *MALAT1*. While the ceRNA model is interesting and *MALAT1* might function as a ceRNA under certain circumstances, more rigorous experiments are needed to prove this model. For instance, the specificity of the siRNAs targeting *MALAT1* or the miRNA targets should be clearly addressed. Moreover, it would be critical to demonstrate that the miRNA-binding site on *MALAT1* is important for its function—key evidence that is generally lacking. In addition, gain-of-function experiments would further strengthen the conclusions. Furthermore, if *MALAT1* functions through sponging multiple miRNAs, it is very challenging to experimentally prove the ceRNA model of *MALAT1* (Figure 2a).

### 5.2. MALAT1 Interacts with the PRC2 Complex

Polycomb repressive complex 2 (PRC2) catalyzes histone H3K27 methylation, which plays important roles in transcriptional repression and cancer [63]. *HOTAIR* was the first reported PRC2-binding lncRNA that recruits PRC2 to target gene loci [64]. Subsequently, it has been shown that *HOTAIR* directly interacts with EZH2 [65], and that *HOTAIR* negatively regulates epithelial gene expression through H3K27 trimethylation [66,67]. Recently, additional lncRNAs, including *MALAT1*, have been shown to bind PRC2 components (Figure 2b). Fan et al. reported that *MALAT1* binds SUZ12, a subunit of the PRC2 complex, and that *MALAT1* and SUZ12 mediate TGF-β induced EMT in bladder cancer [68]. Hirata et al. showed that *MALAT1* interacts with EZH2, the catalytic subunit of PRC2, and that both *MALAT1* and EZH2 are required for the EMT in renal cell carcinoma [69]. In addition, several studies showed that the interaction of *MALAT1* with EZH2 is involved in other cancer types, such as prostate cancer [70], gastric cancer [71], and lymphoma [72]. However, it remains unclear whether and how the *MALAT1*-PRC2 interaction specifically regulates the transcription of target genes. Notably, a recent EZH2 RIP-seq assay identified more than 1,000 EZH2-binding lncRNAs [73], while it is unclear whether and how these lncRNAs regulate the activity of EZH2. Similarly, studies from Cech and colleagues revealed promiscuous RNA binding by PRC2 and indicated that mammalian PRC2 binds thousands of RNAs in vivo [74,75]. While the hypothesis that lncRNAs recruit chromatin-modifying complexes to target gene loci is intriguing, questions remain regarding the molecular mechanism by which lncRNAs regulate transcription.

### 5.3. MALAT1 Binds and Inactivates TEAD

Recently, our group performed a chromatin isolation by RNA purification coupled to mass spectrometry (ChIRP-MS) assay to identify *Malat1*’s endogenous binding proteins in mammary tumors from MMTV-PyMT mice. We identified a list of 23 proteins that specifically bind to *Malat1* but not two negative controls (nuclear RNA *U1* and probe-free beads) [29]. Interestingly, all four members of the Tead transcription factor family (Tead1, Tead2, Tead3, and Tead4) were present in this list. Through subsequent validation by ChIRP-Western, RNA pulldown, RIP-qPCR, and UV crosslinking-immunoprecipitation and qPCR (CLIP-qPCR) assays, we found that *MALAT1* binds to the transactivation domain of TEAD proteins, which are unconventional RNA-binding proteins (RBPs), but does not bind to GAPDH, histone H3, or the TEAD co-activator YAP [29]. Moreover, the results from TEAD reporter assays, co-IP, qPCR, ChIP-qPCR, and functional rescue experiments demonstrated that *MALAT1* lncRNA sequesters the transcription factor TEAD, thereby blocking TEAD from associating with its co-activator YAP and target genes, which in turn leads to inhibition of TEAD’s transcriptional activity and pro-metastatic function in breast cancer [29] (Figure 2c). It remains to be determined whether *MALAT1* suppresses metastasis by inactivating TEAD in other cancer types. In addition, the functional consequences of the interaction of *Malat1* with its other binding partners warrant further studies.

### 5.4. MALAT1 Regulates Multiple Signaling Pathways

In addition to the Hippo-YAP pathway, *MALAT1* has been reported to regulate other signaling pathways in cancer, including PI3K-AKT, MAPK, WNT, and NF-κB pathways. For instance, by modulating Wnt signaling, *MALAT1* has been shown to regulate cancer cell EMT, migration, invasion, and metastasis [76,77,78]. *MALAT1* was also found to regulate hepatocellular carcinoma progression through the mTOR pathway [79]. In certain cancer types, a *MALAT1*–NF-κB axis is involved in chemoresistance and EMT [80,81], and PI3K-AKT signaling has been found to mediate the effect of *MALAT1* on metastasis [30,82,83]. Moreover, *MALAT1* may regulate proliferation and metastasis of esophageal squamous cell carcinoma through the ATM-CHK2 pathway [84]. In addition, *MALAT1* has been reported to regulate tumor cell proliferation through the MAPK pathway [27,36]. Unfortunately, validation of these results in genetically engineered mouse models is lacking, and very little is known about the molecular mechanisms by which *MALAT1* regulates these pathways.

## 6. Conclusions and Future Perspectives

As one of the most abundantly expressed lncRNAs in normal tissues, *MALAT1* has attracted substantial interests from multiple fields including the cancer field. *Malat1* knockout mice are viable and develop normally, suggesting that *MALAT1* is dispensable for development. It remains to be determined whether this lncRNA plays important roles in stress responses or various pathological processes, such as cardiac stress, vascular injury, intestinal injury, immune response, and various oncogenic insults, and whether mice with *Malat1* deficiency show phenotypes in response to external or internal perturbations.

*MALAT1* was previously described by many papers as a cancer-promoting and metastasis-promoting lncRNA, while other reports suggested a tumor-suppressing role of *MALAT1*. A major pitfall in these studies was the lack of rescue experiments for loss-of-function approaches. In contrast, our group conducted genetic rescue experiments to demonstrate that the metastasis induction by *Malat1* germline inactivation or somatic knockout (CRISPR-Cas9) was specific to the loss of *MALAT1* lncRNA [29]. This finding underscores the importance of rigorous characterization of lncRNAs, and illustrates how a lncRNA can interact with unconventional RBPs (TEAD proteins) to inhibit metastasis. Non-coding RNA functions should be unambiguously established by rescue experiments in which the RNA expression is restored in knockout cells or knockout mice by means of an independent transgene. Moreover, it is critical to rule out non-specific effects in all types of loss-of-function experiments, including gene deletion, insertional inactivation, CRISPR-Cas9, RNAi, ASO, and chemical inhibition; this is particularly important for the validation of anticancer targets. The current understanding of lncRNAs remains very limited. Moreover, RNA modifications and RBPs can regulate RNA’s fate; on the other hand, RBPs, especially unconventional RBPs, could be controlled by RNA, as exemplified by the *MALAT1*-TEAD interaction [29]. We still have a lot to learn about lncRNAs and a lot to expect from the discovery of RNA epigenetics and many new unconventional RBPs. The ongoing and future studies will profoundly advance understanding of the roles of RNA biology in tumor progression and metastasis, and will likely unearth novel anti-metastatic targets for treatment.
